# Supplementary material for: Collagen Fibril Density Modulates Macrophage Activation and Cellular Functions during Tissue Repair
Source: Bioengineering (Basel). 2020 Mar 31;7(2):33. doi: 10.3390/bioengineering7020033 (PMC7356036; doi:10.3390/bioengineering7020033)
Supplement: Supplementary file 1 [file bioengineering-07-00033-s001.pdf]

bioengineering

MDPI

Supplementary

Table S1. Primer list.

Gene	Forward	Reverse
RPS26	CAATGGTCGTGCCAAAAAG	TTCACATACAGCTTGGGAAGC
ACTB	CATCCGCAAAGACCTGTACG	CCTGCTTGCTGATCCACATC
aSMA	AGACCCTGTTCCAGCCATC	TGCTAGGGCCGTGATCTC
Coll1a1	GTCGCACTGGTGATGCTG	GGTGGTGTCCACCTCGAG
EDA-FN	CCAGTCCACAGCTATTCCTG	ACAACCACGGATGAGCTG
MHC-II	TCCTGGTCCAACTTCTGTCC	CCCAACCTCATCCGATCTGA
CD163	GAGCAGCACATGGGAGATTG	ACCTCCTCCATTTACCAGGC
CD206	AACGGACTGGGTTGCTATCA	CCCGATCCCTTGTAGAGCAT
IL-10	AGAACCAAGACCCAGACATCAA	AATAAGGTTTCTCAAGGGGCT

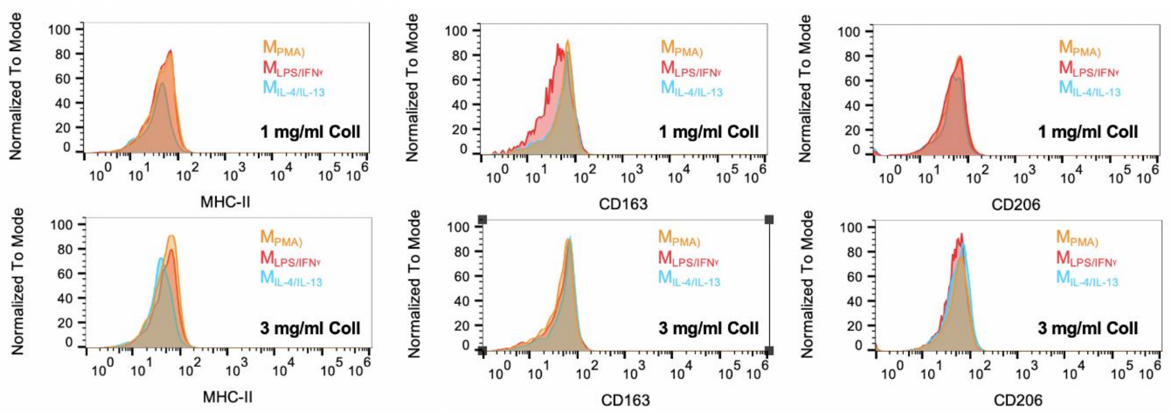

Figure S1. Flow cytometry analysis of unstained macrophages. Representative histogram plot of unstained cells for MHC-II, CD163 and CD206 are shown as a function of fluorescence signal intensity.

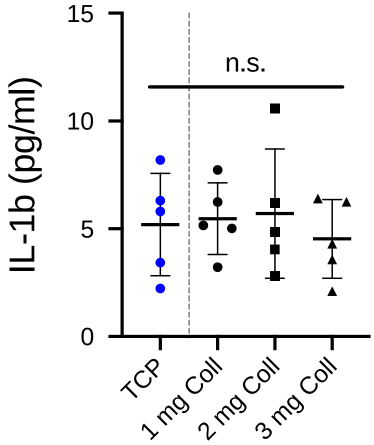

Figure S2. Quantitative analysis of IL-1β secretion by THP-1 cultivated onto tissue culture plastic (TCP) and 3D collagen matrices of concentration of 1, 2 and 3 mg/mL after 3 using ELISA (n = 5).

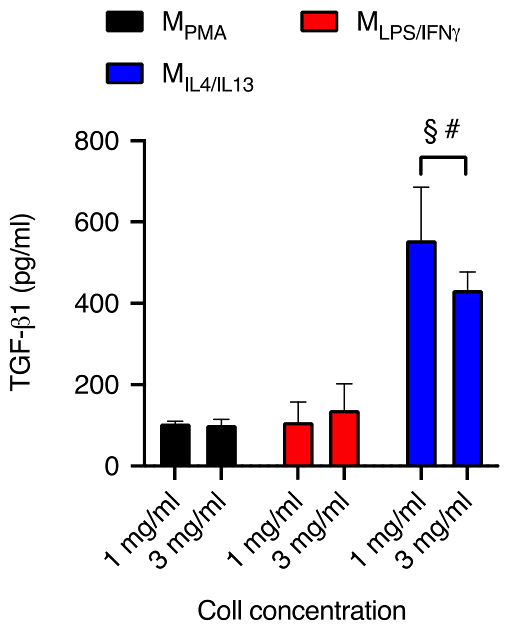

Figure S3. Quantitative analysis of free active TGF-β1 secretion by macrophages in a co-culture with fibroblasts after 3 days of culture using ELISA (n = 4). Data are represented as mean ± SD; * significance level of $p < 0.05$). The characters # and § represent the significance level of $p < 0.05$ when compared to $M0_{PMA}$ and $M1_{LPS/IFN\gamma}$ macrophages, respectively.

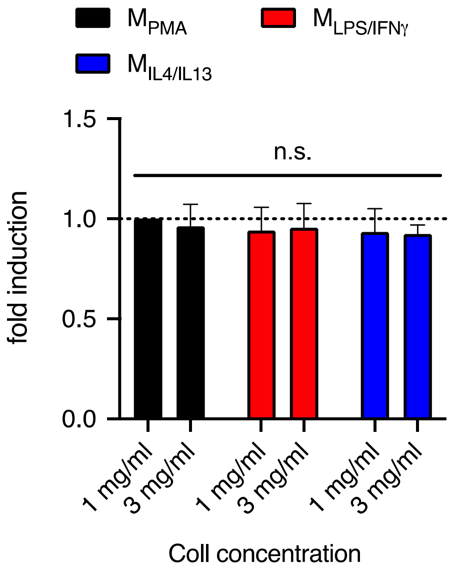

Figure S4. Quantitative analysis of aSMA expression by macrophages after 3 days of culture using qPCR (n = 4). Data are represented as mean ± SD.

© 2020 by the authors. Submitted for possible open access publication under the terms and conditions of the Creative Commons Attribution (CC BY) license (http://creativecommons.org/licenses/by/4.0/).
